# A SIMPLE METHOD FOR THE DIAGNOSIS OF PETERSEN’S HERNIA COMPROMISING THE BILIOPANCREATIC LIMB

**DOI:** 10.1590/0102-672020180001e1429

**Published:** 2019-02-07

**Authors:** Carlos Alberto PERIM, Marcelo Arimatéia Esteves GUEDES, Marcus Flávio Carvalho e CARVALHO, Políbio Guedes Ferreira LOPES, Romeo Lages SIMÕES

**Affiliations:** 1 Saint Luke Hospital, Governador Valadares, MG, Brazil

**Keywords:** Obesit, Bariatric surger, Surgical techniqu, Laparoscopic gastric bypas, Petersen’s herni, Obesidad, Cirurgia bariátric, Técnica cirúrgic, Bypass gástrico laparoscópic, Hérnia de Petersen

## INTRODUCTION

The incidence of internal hernias in laparoscopic Roux-en-Y gastric bypass is 0.5-9.7%^2^ ^,^ ^7^. The diagnosis of intestinal obstruction should always be suspected in the presence of abdominal pain in patients previously submitted to it laparoscopically. Internal hernias are the main causes of intestinal obstruction after this surgical procedure^14^, and may occur through the mesenteric breach at the level of the enteroenteral anastomosis or the Petersen space, located between the transverse mesocolon and the mesentery of the alimentary loop elevated to the gastric pouch via antecolic and antegastric route. The most frequent intestinal obstruction, and also more severe, is that resulting from a Petersen hernia involving the biliopancreatic loop, because it has a closed loop.

In Petersen hernia, the diagnosis can be difficult by a non-specific clinical picture of intestinal obstruction: acute and persistent chronic or intermittent abdominal pain, localized or diffuse; no nausea and vomiting; flatus can continue to be eliminated and the abdomen remains undisturbed^3^. Abdominal examination is usually not relevant. However, the early diagnosis of Petersen hernia is essential for the indication of an emergency operation, preventing severe and even fatal consequences^3^ ^,^ ^10^. The simple abdominal radiographic study is of little value, and computed tomography is the best imaging method to confirm the diagnosis. However, it fails in 20-30% of patients with Petersen hernia^7^. In addition, there is not always the availability of a CT scanner or qualified professional for the interpretation of the exam, and the surgeon remains uncertain and responsible to define what to do.

Thinking about these difficulties we used a rather simple method, in an attempt to confirm the early diagnosis of the Petersen hernia involving the biliopancreatic loop.

The method is based on normal anatomy, in which the jejunal loop closest to the duodenojejunal angle is always located to the left of the spine, and after laparoscopic gastric bypass, the only way the jejunal segment passes to the right side of the spine is through the space of Petersen.

## METHOD

**FIGURE 1:**
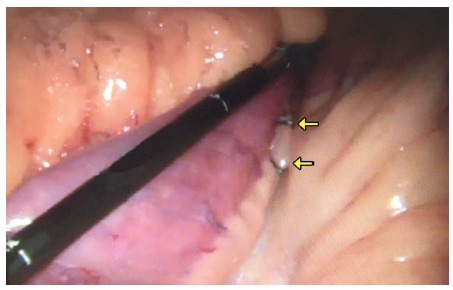
Two clips attached to the biliopancreatic loop mesentery 10 cm from the duodenojejunal angle and to the left of the spine

In laparoscopic gastric bypass the initial step consists of an inventory of the jejunal loops, and at this time two metallic clips are applied to the mesentery, 1 cm apart and 10 cm from the duodenojejunal angle (Figure 1).

**FIGURE 2:**
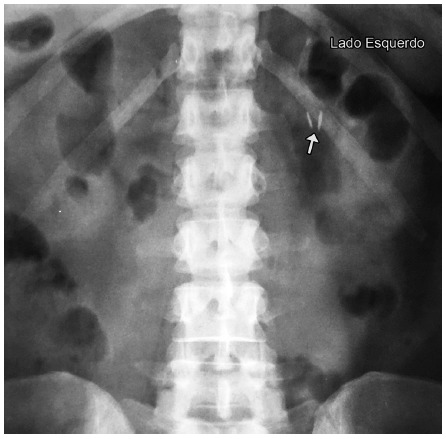
Simple abdominal radiographic study revealing the two clips in expected normal position: to the left of the spine

Clips attached to the mesentery will always be located to the left of the spine and will be easily seen on a simple abdominal radiograph (Figure 2).

**FIGURE 3:**
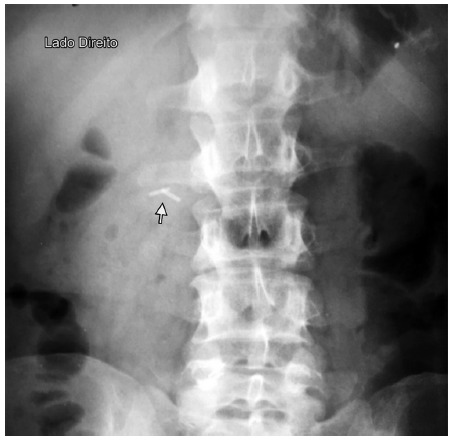
Simple abdominal radiographic study showing the two clips on the right side of the spine. In this case, the diagnosis of Petersen hernia was confirmed in laparoscopy

In cases of Petersen hernia, the biliopancreatic loop, when sliding behind the food loop, carries the clips, which will then be positioned to the right of the spine, and a simple abdominal radiograph confirms the diagnosis (Figure 3)

## CASE REPORT

In the period from February 2016 to December 2017, 165 patients underwent laparoscopic Roux-en-Y gastric bypass, in which we applied two metal clips in the jejunum mesentery, 10 cm from the duodenojejunal angle. The Petersen space was not closed in 142 patients (86%) and closed in the last 23 (13.9%).

Among these 165 patients, in one was recognized Petersen hernia, ie, clips to the right of the spine. The diagnosis was made by simple x-ray of the abdomen (Figures 3). The others had the clips systematically to the left of the spine.

## DISCUSSION

The proposed method is based on normal anatomy, in which the jejunal loop near the duodenojejunal angle is always located to the left of the spine (Figure 2). After laparoscopic gastric bypass, the only way this jejunal segment can passes to the right side of the spine is through the Petersen space. When the Petersen hernia occurs, the biliopancreatic loop, as it slides behind the food loop, carries the clips, which are then positioned to the right of the spine (Figure 3).

There is a current consensus that all mesenteric spaces should be closed in laparoscopic Roux-en-Y gastric bypass, which reduces the incidence of internal hernias^15^. However, in many patients Petersen’s closure of the space is not or is not yet done, due to the greater technical difficulty. The closure of this space is not free of complications and may reopen^6^ ^,^ ^8^. Hernias that occur through orifices originating in the suture line for the closure of the Petersen space are more prone to intestinal ischemia than those occurring when the space is wider when not closed. In any circumstance, when biliopancreatic loop obstruction occurs, clinical evolution is potentially catastrophic, because it is a closed loop with a more rapid evolution for intestinal necrosis. 

The fixation of the biliopancreatic loop to the transverse mesocolon, in order to prevent the occurrence of Petersen hernia, is the only alternative proposal to the closure of the Petersen space described in the literature^12^. However, to our knowledge, there isn´t no medical evidence of the efficacy of this technique.

**FIGURE 4:**
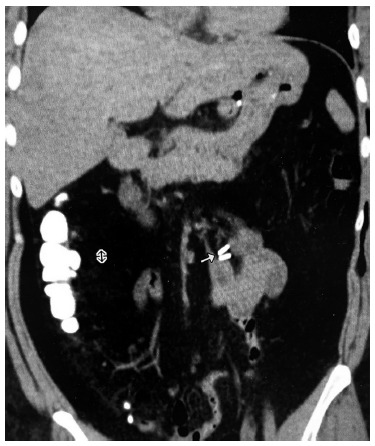
Abdominal CT scan: clips in normal position, to the left of the spine in case of obstruction in the anastomosis, not Petersen’s hernia, which regressed with conservative treatment (control CT revealing contrast in the ascending colon).

Regardless of the closure or not of the Petersen space, hernias can occur through this space at any time in the postoperative period of the laparoscopic gastric bypass, and the important thing in this circumstance is the early diagnosis. This is what the method described above is intended to ensure. Needing two metal clips applied to the mesentery to be performed, does not cause risks to the patients, practically does not increase the operative time and is of negligible cost. If the option is CT, the image of the clips will be equally prominent and easy to see, even by those who have no experience in interpreting this exam (Figure 4). 

**FIGURE 5:**
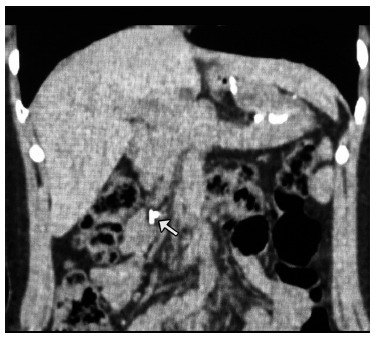
Abdominal CT interpreted as normal. However, subsequent surgical review revealed clips to the right of the spine, signaling Petersen’s hernia

In the simple abdominal radiographic study, the clips will be located to the right of the spine when there is Petersen hernia (Figure 5).

The method outlined will be especially valuable in the evaluation of pregnant women with suspected Petersen hernia^16^ when a simple radiograph limited to the upper abdomen will allow the diagnosis with much less irradiation load than the tomography.

In the occurrence of Petersen hernia in patients submitted to previous laparoscopic cholecystectomy, the distinction between the clips fixed in the duct and cystic artery and the mesenteric clips will be made by the sum and position of the clips, besides the absence of clips to the left of the spine.

Intestinal obstructions after gastric bypass in laparoscopic Roux-en-Y may occur more rarely in several other causes - adhesions, clots and intussusceptions - and compromise other intestinal segments, including the alimentary loop^1^ ^,^ ^4^. In these eventualities, if there is no concomitant involvement of the biliopancreatic loop, the recommended method will not allow diagnosis because the clips will remain to the left of the spine. Regardless of the results of any imaging examination, the surgeon should maintain a high degree of suspicion and not hesitate to indicate emergency operation in cases of persistent abdominal pain with no established cause after laparoscopic Roux-en-Y gastric bypass^13^.
